# Indicators of home-based hospitalization model and strategies for its implementation: a systematic review of reviews

**DOI:** 10.1186/s13643-020-01423-5

**Published:** 2020-08-08

**Authors:** Christiane Pereira Martins Casteli, Gisèle Irène Claudine Mbemba, Serge Dumont, Clémence Dallaire, Lucille Juneau, Elisabeth Martin, Marie-Claude Laferrière, Marie-Pierre Gagnon

**Affiliations:** 1 Faculty of Nursing Sciences, Université Laval, Québec City, QC Canada; 2 University Health and Social Services Centre (IUHSSC) of Capitale-Nationale (CN), Québec City, QC Canada; 3 School of Social Work, Université Laval, Québec City, QC Canada; 4 Primary Care and Services Research Center, Université Laval - Primary Health Care and Social Services University Institute, IUHSSC-CN, Québec City, QC Canada; 5 Research Center of the CHU de Québec-Université Laval, 1050 Avenue de la Médecine. Pavillon Ferdinand-Vandry, Québec City, QC G1V0A6 Canada; 6 Center of Excellence on Aging Quebec (CEVQ), IUHSSC-CN, Québec City, QC Canada; 7 Library, Université Laval, Québec City, QC Canada

**Keywords:** Home-based hospitalization, Home hospital, Home care, Systematic review, Meta-analysis

## Abstract

**Background:**

Home-based hospitalization (HBH) offers an alternative delivery model to hospital care. There has been a remarkable increase in pilot initiatives and deployment of this model to optimize services offered to a population with a variety of progressive and chronic diseases. Our objectives were to systematically summarize the indicators of HBH as well as the factors associated with the successful implementation and use of this model.

**Methods:**

We used a two-stage process. First, five databases were consulted, with no date delimitation. We included systematic reviews of quantitative, qualitative, and mixed studies published in English, French, Spanish, or Portuguese. We followed guidance from PRISMA and the Cochrane Collaboration. Second, we used the Nursing Care Performance Framework to categorize the indicators, a comprehensive grid of barriers and facilitators to map the factors affecting HBH implementation, and a thematic synthesis of the qualitative and quantitative findings.

**Results:**

Fifteen reviews were selected. We identified 26 indicators related to nursing care that are impacted by the use of HBH models and 13 factors related to their implementation. The most frequently documented indicators of HBH were cost of resources, problem and symptom management, comfort and quality of life, cognitive and psychosocial functional capacity, patient and caregiver satisfaction, hospital mortality, readmissions, and length of stay. Our review also highlighted new indicators, namely use of hospital beds, new emergency consultations, and use of healthcare services as indicators of resources of cost, and bowel complications, caregiver satisfaction, and survival time as indicators of change in the patient’s condition. The main facilitators for HBH implementation were related to internal organizational factors (multidisciplinary collaboration and skill mix of professionals) whereas barriers were linked to the characteristics of the HBH, specifically eligibility criteria (complexity and social situation of the patient).

**Conclusion:**

To the best of our knowledge, this is the first review that synthesizes both the types of indicators associated with HBH and the factors that influence its implementation. Considering both the processes and outcomes of HBH will help to identify strategies that could facilitate the implementation and evaluation of this innovative model of care delivery.

**Systematic review registration:**

PROSPERO CRD42018103380

## Background

With the aging population and the growing prevalence of chronic diseases affecting all age groups, the integration of home care services is becoming a necessity for front line health service organizations around the world [1–3]. Since hospitalization is one of the key factors in the increasing cost associated with the use of health services related to chronic diseases, it is essential to implement effective and safe alternatives to conventional hospitalization [4].

All over the world, emergency room overflows reflect on suboptimal performance of the healthcare system as it is currently organized and delivered [5–7]. In addition, hospitalization in emergency departments entails significant risks for older adults, including iatrogenic complications, functional and cognitive decline, and loss of independence [8, 9].

Home-based hospitalization (HBH) offers an alternative model of care delivery subject to the same obligations as hospitals [10, 11]. The terminology regarding this service model is inconsistent in the literature as many studies use hospital-based home, hospital at home, hospital in the home, and home-hospitalization. In some cases, these terms are used but do not involve substitution for in-hospital care. The operational definition for HBH that we adopt in this paper is a service that provides in-home hospital care to patients with complex clinical conditions who would be hospitalized in conventional facilities due to an acute episode [12] and require 24/7 monitoring and follow-up that is only available in the hospital [13]. The implementation of HBH would therefore optimize the use of resources by providing health services for specific groups who do not require conventional hospitalization.

In this regard, several countries, including the USA [14], Spain [15], Australia [16], Canada [17], and the UK [18], have implemented HBH, following the example of France which was one of the first jurisdictions to implement it [19, 20]. The criteria for home admission are very heterogeneous, and the activity of HBH care varies greatly according to the main management methods: complex dressings, palliative care, and intensive nursing interventions [21].

A brief summary of Cochrane systematic reviews and meta-analyses comparing conventional hospitalization and HBH reveals that HBH would substantially optimize hospital bed [22] and would have a small advantage in readmissions [12, 20, 23] and patient satisfaction [22, 24, 25]. However, there was no significant difference between the two modalities of care in terms of cost (reduced length of stay in hospital) or improved health outcomes, including reduced mortality [20, 22–24, 26].

The heterogeneity between systematic reviews reveals the varying degree of structuring of home care services with respect to the characteristics of the population and organization of services, the measures used, and the results reported [12, 27]. Thus, it becomes a challenge to find the most advantageous model. Despite the growing interest in HBH models, their implementation is still difficult for countries that do not have national and federal standards governing this practice [9, 13]. In general, arrangements for organizing HBH services, team composition, and organization of health professionals, as well as patient care and follow-up visits, are not well defined in the studies and precluding firm conclusions [12, 27, 28]. This limits the possibility to understand how organizational, clinical, economic, political, and social factors influence the implementation of this model of care.

The lack of knowledge about implementation factors compromises the identification of the expected effects in the delivery of HBH care and services. It also limits knowledge transfer about the organizational structure required to adopt this model of care between countries that have a comparable health system.

## Why do this systematic review of reviews?

Several systematic reviews and meta-analyses have assessed a wide range of indicators related to HBH, including the cost of providing hospital-based home care compared to conventional hospitalization. However, these indicators are not organized into a structured framework and their measurement vary between studies, which makes difficult providing clear evidence of the effects of HBH on important outcomes. Moreover, no previous reviews have systematically synthesized evidence on the factors associated with the implementation of HBH models. Indeed, the implementation of a new model of care is synonymous with major changes and transformations in the organization of services as well as at the clinical, economic, political, and social levels [29, 30].

This synthesis highlights the facilitating and limiting factors for HBH implementation together with its indicators, thus contributing to the knowledge base regarding this innovative model of care delivery for healthcare organizations.

## Objectives

Systematically mapping the indicators of HBH as well as the factors associated with the success of the implementation and use of this model of care.

More specifically, this review covers the following questions: (1) What barriers and facilitating factors influenced the implementation of the HBH model? (2) What indicators (positive, negative, or neutral indicator) have been used to measure the HBH model?

## Methods

### Study design

As there are currently no guidance on reporting systematic reviews of reviews, we used the “Preferred Reporting Items for Systematic Review and Meta-Analysis” extension for network meta-analyses (PRISMA-NMA) guidelines [31] as a general framework to report this work. We also consulted methodological references on overviews of systematic reviews [32–34]. The PRISMA-NMA guidelines which contains 27-item checklist and a four-phase flow diagram.

This review was structured according to the formulation of the PICOS research question (Population, Intervention, Comparison, Outcomes, Study Designs) [32] which forms the basis for establishing the components and eligibility criteria for studies (Table 1). Our PROSPERO protocol indicates the population of patients with chronic diseases, more specifically in palliative care because it was the target population of the larger project. However, we decided during the elaboration of the research strategy to expand the population including also patients in acute conditions in order to favor the inclusion of different models of HBH.

**Table 1:** Definition of PICOS criteria for the eligibility of studies

**Population**	Patients with chronic diseases, acute conditions, or in palliative care
**Intervention**	Home-based hospitalization (HBH)
**Comparaison**	Conventional hospital care
**Outcomes**	**Primary:** indicators of HBH use on access, continuity, quality, and safety of care, clinical practices, organization of health services, costs at the patient, family, health system, and society levels **Secondary:** facilitating factors and barriers to the implementation of HBH at the environmental, organizational, staff, patient, and family levels.
**Study designs**	Systematic review of quantitative, qualitative, or mixed-methods studies, with or without meta-analysis

The methodological quality assessment grid for systematic reviews (AMSTAR 2) was used for assessing the quality of systematic reviews and meta-analyses included [35].

The nursing care performance framework (NCPF), developed by Dubois [36] (Appendix 1), has been adapted to map the indicators of HBH identified from systematic reviews. This model allows the systematic evaluation of the healthcare system in general, including nursing care, and its three subsystems (acquiring, maintaining, and deploying resources; transforming resources into services; producing changes in patient conditions). It proposes 14 dimensions and 51 indicators that allow performance evaluation of the whole nursing system using a multidimensional perspective that includes structure, process, and results and takes into account the influence of external factors.

The NCPF was chosen because in the HBH model, the coordination of care is centered on nursing practice [11, 37–39]. This model provides a group of indicators to evaluate nursing performance in the organizational model of HBH that could serve as a basis to orient evaluation of this model of care.

Regarding the factors that influenced the implementation of the HBH model, we adapted the concepts developed through research related to the classification of barriers and facilitators to implementing innovative technologies in healthcare [40–43]. This conceptual approach allows an explanation of the factors affecting the implementation and use of the HBH model in a complex and dynamic environment inherent to the healthcare system and defines the determinants of the adoption and diffusion of this innovation.

### Protocol

The protocol for this review is registered in the international prospective register of systematic reviews (PROSPERO) under number CRD42018103380. We brought some changes to the protocol as we did not limit the population to HBH for palliative care, but rather included all populations that could benefit from HBH, since the included systematic reviews often considered several population groups or diseases. We also considered all relevant outcomes reported in the included systematic reviews.

### Inclusion and exclusion criteria

Included publications were quantitative systematic reviews (including meta-analysis), qualitative reviews, and mixed studies reviews focusing on the factors associated with the implementation of HBH and its indicators of use, and published in English, French, Spanish, and Portuguese (languages spoken by the authors). As the first systematic review on HBH was published in 1998 [44], we did not delineate date limit for the search.

### Search strategy

Five electronic databases (Medline (OVID), Embase, Cochrane Database of Systematic Reviews (CDSR), Database of Abstracts of Reviews of Effects (DARE), and CINAHL Plus with Full Text bibliographic databases using controlled and free terms) were searched between April and May 2018 and October 2019 (Appendix 2). The development of the search strategy in all selected bibliographic databases was carried out by two team members (CPMC, MCL) as the latter is a health librarian. The SIGN [SIGN] search filter was used by the specialist (MCL) to specify the research process with a predefined set of keywords to identify systematic reviews and meta-analyses. The results of each search were recorded in a bibliographic reference management software (EndNote). Duplicate references were eliminated.

### Selection of studies

The selection was made independently by two team members (CPMC, GICM). First, the titles and summaries of the systematic reviews were reviewed and selected according to the inclusion criteria. Then, the complete texts were evaluated. Publications that did not meet the inclusion criteria were excluded by documenting the reasons for exclusion. Any disagreement concerning study eligibility was resolved through discussion and consensus involving both examiners or involved a third author, if necessary. A flowchart was used to show the overall process of selecting studies [45].

### Extraction of data

Data from the included reviews were extracted independently by two team members (CPMC, GICM), as recommended by the Cochrane Handbook [32], using a form based on the components of the PICOS question and primary and secondary outcome indicators. The following data were extracted: characteristics of the review (authors, year of publication, language, type of review, rationale, objectives), characteristics of the population (patient profile, health status, and care environment), characteristics of the intervention (type of service provided, context of care, duration of the HBH, intervention components, team composition, technologies used), comparisons between home and conventional hospitalization, primary and secondary outcomes (positive, negative, or neutral indicators of HBH and implementation determinants). For summarizing our qualitative findings, two team members (CPMC, GICM) adapted the grid with concepts developed through research related to the classification of barriers and facilitators to implementing innovative technologies in healthcare [40–43] as the analytical framework. CPMC and GICM independently performed data extraction and transposed extracted data into this framework using thematic analysis. We populated the data extraction grid in the Microsoft Excel software 2016. The quality of included reviews was independently assessed by the same two authors according to the AMSTAR 2 evaluation grid [35]. The strength of the evidence was assessed according to the GRADE approach [46].

### Data synthesis

A narrative summary of the results of the included reviews was developed to describe the main indicators of HBH using the framework of Dubois [36]. Implementation factors were also synthesized narratively using the categories proposed by McGinn et al. [40] and Gagnon et al. [43]. The narrative approach is recommended to summarize and explain the results of systematic quantitative, qualitative, and mixed studies, especially the indicators of interventions and the implementation of interventions that have been shown to be effective [47, 48].

Specifically on implementation factors, the reviewers identified sections of the publications that presented a relevant barrier or facilitator to implementation and use of HBH model and coded them according to the categories proposed in the grid. Then, we grouped the extracted data into four main categories of adoption factors, and each category was decomposed into specific factors.

To achieve this, quantitative data were categorized according to the frequency of each factor and its influence (facilitator or barriers as well as the frequency of each indicator (positive, negative, or neutral indicators) of using HBH at the level of patient, family, healthcare system, and society. Qualitative data were integrated into a thematic synthesis.

## Results

### Search results

**Fig. 1:**
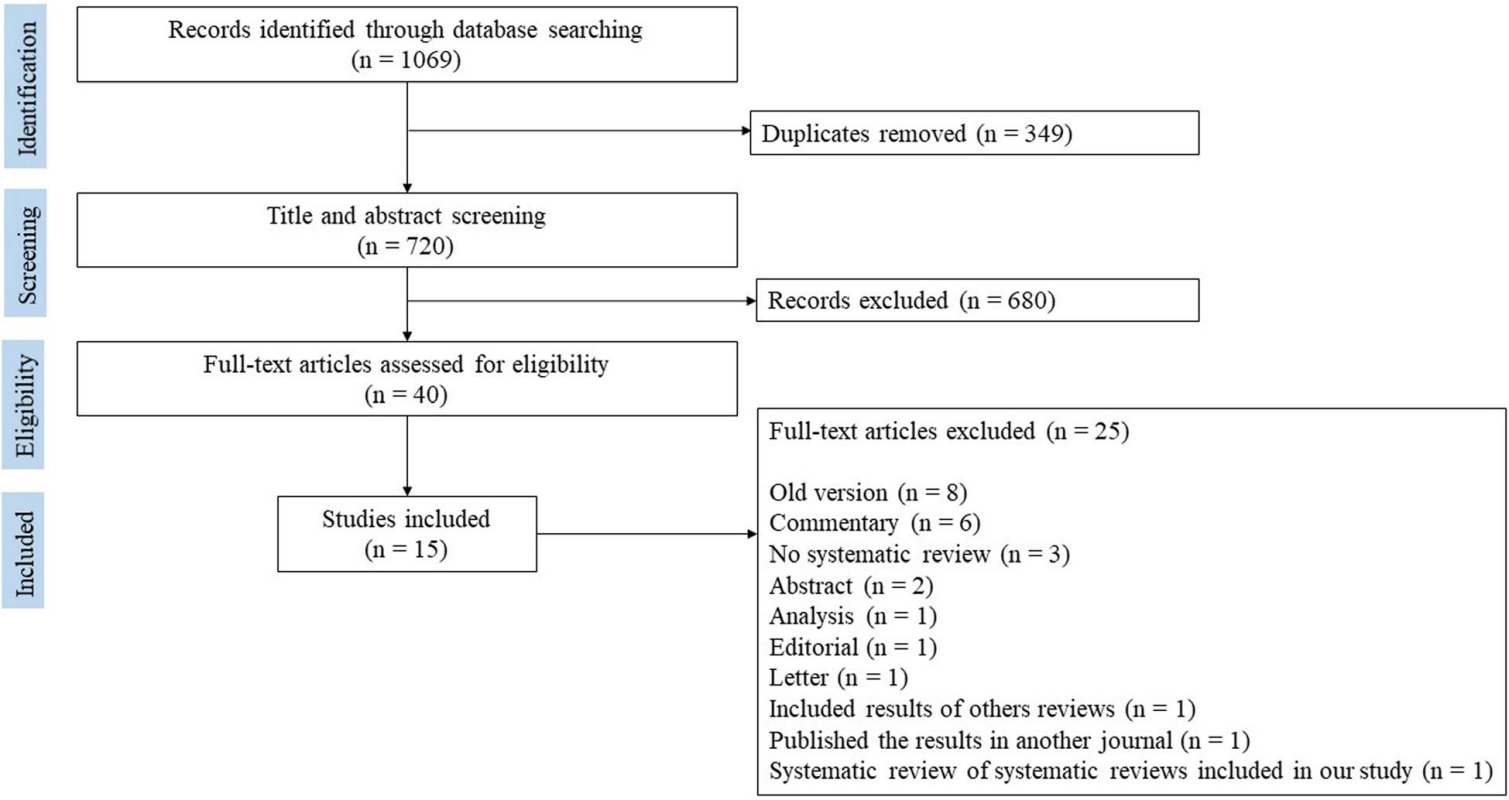
PRISMA study flow diagram

A total of 1069 records were identified from the search strategy. After removing duplicates and screening titles and abstracts, we examined 40 full texts, of which 15 reviews [12, 20, 22–26, 44, 49–55] were eligible for inclusion. All reviews showed indicators of HBH as well as the factors associated with the implementation and use of this model of care (by primary and secondary outcomes). However, the structure of included reviews did not allow us to present the outcomes by population groups. Appendixes 3 and 4 present the PRISMA extension checklist and the list of selected reviews with the frequency of primary studies, respectively. The overall process of review selection was summarized according to the PRISMA study flow diagram (Fig. 1), and details are provided regarding the primary reasons for exclusion and the full references of excluded publications (Appendix 5).

### Characteristics of the reviews

The general characteristics of the included reviews, namely type of review, population, intervention, outcomes, and quality, are summarized in Table 2 and detailed in Appendix 6.

**Table 2:** Summary of characteristics of included studies

Author, year, country	Type of reviews or designs	Population	Intervention	Outcomes	AMSTAR
**Cool et al., 2018** [53], Belgium	Systematic review Mixed	Adult patients	Parenteral cancer drug administration	Quality of life, patient’s satisfaction, safety, and costs	Moderate
**Corral Gudino et al., 2017** [54], Spain	Systematic review Qualitative	Not specified	Interventions supporting continuity of care, including HBH	Number of readmissions, mortality, or improvement in functional capacity	Moderate
**Goncalves-Bradley et al., 2017** [22], UK	Systematic review and meta-analysis Quantitative	Patients aged 18 years and over	Early discharge hospital at home	Effectiveness and cost of the intervention	High
**Huntley et al.** [55], **2017**, UK	Systematic review Qualitative	Patients aged over 65 years	Any community-based intervention offered as an alternative to admission to an acute hospital	Reduction in secondary care use, patient-related outcomes, safety, and costs	Moderate
**Shepperd et al., 2016a** [24], UK	Systematic review and meta-analysis Quantitative	People aged 18 years and older	Home-based end-of-life care	Place of death, admission to hospital, patient satisfaction, caregiver burden, health service costs.	High
**Shepperd et al., 2016b** [25], UK	Systematic review and meta-analysis Quantitative	Patients aged 18 years and over.	Hospital at home	Mortality, transfer to hospital, place of residence, length of stay, patient satisfaction, cost	High
**Echevarria et al., 2016** [23], UK	Systematic review and meta-analysis Quantitative	Patients with acute exacerbation of chronic obstructive pulmonary disease	Early supported discharge (ESD) and hospital at home (HAH)	Readmissions, mortality, and cost.	Moderate
**Qaddoura et al., 2015** [49], Canada	Systematic review and meta-analysis Quantitative	Patients who required hospitalization for decompensated heart failure	Substitutive care models	Mortality, hospital readmissions, other clinical, patient-centered, and cost outcomes	High
**Caplan et al., 2012** [20], Australia	Systematic review and meta-analysis Quantitative	Patients aged > 16 years	Hospital at home care models	Mortality, readmission rates, patient and carer satisfaction, and costs	Moderate
**Jeppesen et al., 2012** [12], Norway	Systematic review and meta-analysis Quantitative	Patients with a diagnosis of COPD with an acute exacerbation	Hospital at home care	Readmission rate, mortality, costs, and days of care provision	High
**Hansson et al., 2011** [50], Denmark	Systematic review Quantitative	Children and adolescents aged 0–18 years with a cancer diagnosis	Care in the patient’s own home as an alternative to a hospital admission	Children’s physical health and adverse events, satisfaction and quality of life of children and their parents, and costs	Low
**Shepperd et al., 2009** [51], UK	Systematic review and meta-analysis Quantitative	Patients aged 18 years and over	Early discharge hospital at home	Mortality, readmissions, patient satisfaction, length of stay in hospital and hospital at home, cost	Moderate
**Shepperd et al, 2008** [52], UK	Systematic review and meta-analysis Quantitative	Patients aged 18 years and older	Hospital care at home	Mortality, readmissions or transfers to hospital, patient and caregiver satisfaction, place of residence at follow-up, length of stay, and cost	Moderate
**Felix et al., 2004** [26], UK	Systematic review and meta-analysis Quantitative	Adult patients	Hospital at home schemes	Mortality and readmission	Moderate
**Shepperd et al., 1998** [44], UK	Systematic review Quantitative	Patients aged 18 years and over	Hospital at home care	Mortality, re-admissions, costs, patient satisfaction, and carer satisfaction	Low

All reviews were published in English, except one that was published in Spanish, between 1998 and 2018, and more than half were published since 2012. The majority of reviews were from the UK (*n* = 9, 60%). The other six reviews were from Canada (*n* = 1), Australia (*n* = 1), Norway (*n* = 1), Spain (*n* = 1), Belgium (*n* = 1), and Denmark (*n* = 1).

The majority of reviews included only quantitative studies (*n* = 12, 80%) [12, 20, 22–26, 44, 49–52]. Three reviews (20%) also included qualitative studies [53–55].

The most common population included in HBH programs were adult patients [12, 20, 22–26, 44, 49, 51, 52]. Only one review assessed the impact of hospital-based home care (HBHC) on children with cancer [50].

According to the AMSTAR 2, five of the reviews were of high quality [3, 22, 24, 25, 49], eight reviews were of moderate quality [20, 23, 26, 51, 52, 55], and two reviews had a low quality score [44, 50].

### Profile of admitted patients in HBH

Fourteen reviews included patient populations aged 18 years and older needing treatment during an acute episode of care, who would otherwise require hospitalization [12, 20, 22–26, 44, 49–55]. The other review focused on children and adolescents aged 0–18 years old [50].

Most reviews considered the provision of HBH to patients with a mix of medical conditions (*n* = 12; 80%), including chronic obstructive pulmonary disease (COPD), stroke, heart failure, elective surgery, pneumonia, psychiatric disease, pulmonary embolism, complicated diverticulitis, and cellulitis [12, 20, 22, 23, 25, 26, 44, 49, 51, 52, 54, 55]. Two reviews included exclusively patients with cancer under chemotherapeutic treatment [50, 53], and one review focused on end-of-life patients [24].

### Overview of the indicators of HBH

**Fig. 2:**
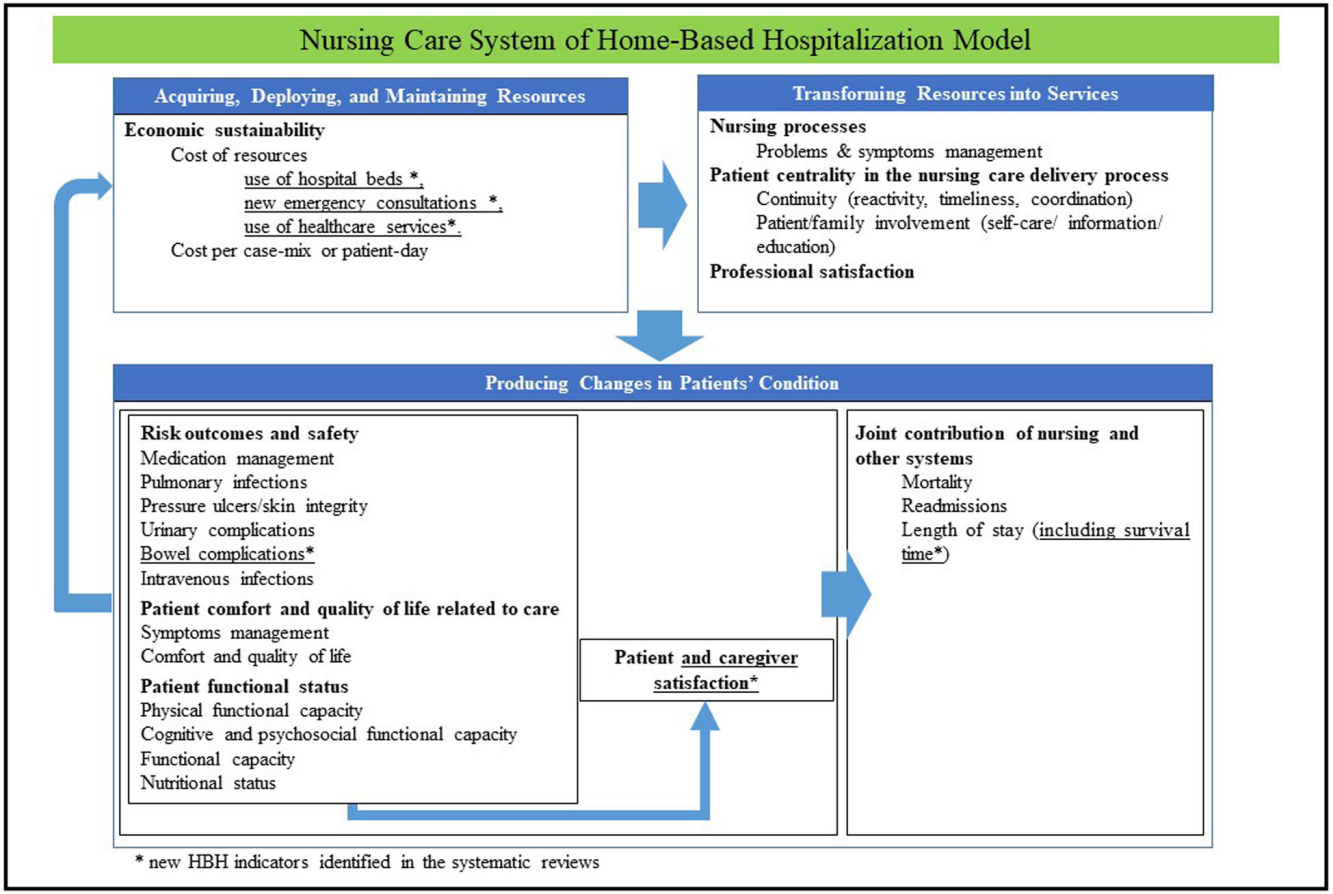
Presentation of results about indicators of HBH model

The results (see Fig. 2) are presented in association with the NCPF [36]: the function, the dimension, and the indicators, according to the frequency of data extracted from each systematic review (Table 3) and with the strength of evidence of according to the GRADE [46] approach identified in the systematic reviews that used it (Appendix 7).

**Table 3:** Frequency and direction of reported indicators of HBH according to the NCPF

Subsystems, dimensions, and indicators from the NCPF	No. of systematic reviews and meta-analyses		
	Positive indicators of HBH	Negative indicators of HBH	Neutral indicators of HBH
**1. Acquiring, deploying and maintaining nursing resources**			
**Economic sustainability**			
Cost of resources	9	-	5
Use of hospital beds*	1	-	-
New emergency consultations*	2	-	-
Use of healthcare services*	1	‑	-
Cost per case-mix or patient-day	1	-	-
**2. Transforming nursing resources into relevant nursing services**			
**Nursing processes**			
Problem and symptom management	3	-	1
**Patient centrality in the nursing care delivery process**			
Continuity (reactivity, timeliness, coordination)	1	-	-
Patient/family involvement (self-care/information/education)	1	-	1
**Professional satisfaction**	2	1	-
**3. Producing changes in patients’ conditions**			
**Risk outcomes and safety**			
Medication management: errors and complications	-	1	2
Pulmonary infections	-	1	-
Pressure ulcers/skin integrity	-	1	-
Urinary complications	1	-	-
Bowel complications*	1	**-**	-
Intravenous infections	-	2	-
**Patient comfort and quality of life related to care**			
Symptom management (e.g., pain, nausea, dyspnea, fever)	3	-	-
Comfort and quality of life (taken broadly)	5	-	3
**Patient functional status**			
Physical functional capacity	-	1	-
Cognitive and psychosocial functional capacity	5	-	1
Functional capacity	1	-	4
Nutritional status	2	-	-
**Patient and caregivers satisfaction**			
Patient satisfaction/complaints	11	1	2
Satisfaction of caregivers and complaints*	4	2	3
**Joint contribution of nursing with other care**			
Hospital mortality	5	-	7
Readmissions	4	-	7
Length of stay	6	1	2
Survival time*	-	-	1

*New HBH indicator identified in the systematic reviews and meta-analyses and integrated to the NCPF

### Function 1: acquiring, deploying, and maintaining resources

#### Economic sustainability

Almost all reviews (14/15) outlined positive indicators related to cost effectiveness of resources [12, 20, 23–26, 49, 52–55], including the three new indicators identified in the HBH economic sustainability dimension of the NCPF, namely use of hospital beds [24], new emergency consultations [49, 54], and use of healthcare services [24]. Only one review did not evaluate the cost [54]. The cost per episode of healthcare or per day basis associated with HBH was consistently lower than usual care [12, 20, 23, 24, 52, 53, 55], and there could also be a saving of bed days a year, according to one review [26].

However, this benefit is offset when costs from a societal perspective were also considered, including formal and informal carer costs and production losses for the patient, over the acute and follow-up periods combined [20, 23, 24, 52]. Some evidence indicates the possibility of substantial variation in the actual effect size [12, 22, 44, 49–51] by patient eligibility criteria [20, 52], different countries, and various conditions [12].

Due to different methods to calculate costs and heterogeneity due to different currencies and different cost structures, identifying the cost-effectiveness of HBH model was not possible in some reviews [22, 44, 49–51].

### Function 2: transforming resources into services

#### Nursing processes

Three reviews found a positive indicator of HBH on problem and symptom management [22, 49, 55] whereas one review found neutral indicator [12]. Among the documented outcomes, HBH reduced delirium [22] and provided improvements for depression [49, 55] and nutritional status at 6 months follow-up [49].

#### Patient centrality in the nursing care delivery process

One review that documented continuity (reactivity) for patients and patient/family involvement (self-care/information/education) showed a positive indicator of HBH [23]. This review found that patient and carer education for recognition and management of acute exacerbation was associated with a lower rate of all-cause readmission.

In relation to informal caregivers, also considered users of this model of care, a review does not report any indicator of the HBH model on them, justifying the lack of data on the impact of home hospitalization on the family or informal caregivers [22]. This review indicates that the caregiver’s willingness to take on the responsibilities associated with HBH is a determining factor that may restrict the degree to which HBH model can be implemented, and this may in turn impact on how these services reduce costs and reliance on secondary care in general [22].

#### Professional satisfaction

Professional satisfaction was reported in three reviews [22, 25, 51]. Indicators of professional satisfaction were mixed within and across the reviews: two reviews demonstrated positive indicators [22, 51], and one demonstrated negative indicator [25].

Two reviews showed that the HBH staff perceived that providing care in the patient home facilitated participation in rehabilitation, that the service was better staffed than the usual discharge services provided, although the workload was similar to conventional hospitalization [22, 51]. However, the evaluation of health professionals’ perceptions about HBH, specifically that of general practitioners, presented limitations due to low response rate [25], although it was higher when compared to the response rate of conventional hospital staff [25].

### Function 3: producing changes in patients’ condition

#### Risk outcomes and safety

Three reviews found negative indicators [25, 50, 53], two reviews found neutral indicators [53, 55], and one found positive indicators [25] on risk outcomes and safety.

The negative indicators included intravenous infections due to perfusion difficulties for which hospitalization was needed, device-related infection [53], and episodes of subcutaneous inflammation along intravenous lines [50]. Pressure ulcers/skin integrity was mentioned in the risk of advancing cellulitis in participants with cellulitis, and pulmonary infections were related to the increased antibiotic therapy for participants with COPD allocated to HBH [25]. Also, the need for management of drug administration was cited before the occurrence of occlusion of central venous catheters [50]. Nonetheless, two reviews mentioned that they found neutral indicators on medication management and that the adverse events described (number of reported toxicities, perfusion difficulties, missed doses, and adverse drug reactions) were comparable between HBH and usual care [53, 55].

The only review that found positive indicators cited the reduction in the number of urinary and bowel complications in patients allocated to HBH [25], the latter being a new indicator identified in the risk outcomes and safety dimension of the NCPF.

#### Patient comfort and quality of life related to care

Positive indicators on symptom management were reported in three reviews [25, 49, 50]. The control of symptoms in adult patients (pain, nausea/vomiting, constipation, diarrhea, breathlessness, anxiety, and depression) improved, but assessments varied by assessor [25]. Fewer patients with stroke allocated to HBH reported anxiety [49]. The successful control of nausea and vomiting in children was also highlighted [50].

Positive indicators on the quality of life (QoL) of patients in HBH were reported in five reviews [25, 49–51, 55], and other three reviews found no association between HBH and health-related quality of life indicators [12, 23, 53]. The improvement in QoL among HBH patients may be facilitated by treatment in a familiar environment, with greater independence and less technically oriented care [49]. Furthermore, HBH presented a significantly reduced risk to patients for being in residential care at follow-up [51]. An improvement was reported in QoL at both 6 months [25, 55] and 12 months, for patients with heart failure [55]. QoL in children and parents was overall improved when the child received intravenous chemotherapy at home with HBH [50].

#### Patient functional status

Five reviews found positive indicators in view of patient functional status [25, 49, 51, 52, 55], five found neutral indicator [22, 24, 25, 51, 52], and one found a negative indicator [55].

HBH patients experienced improvements in cognitive and psychosocial functional capacity with regard to depression [49, 52, 55], especially in patients with stroke or acute chronic heart failure [25], and better psychological well-being for patient with stroke [51]. Fewer participants with a mix of conditions receiving HBH care experienced short-term confusion during an episode of care, and fewer participants with dementia were prescribed antipsychotic drugs [25] or had problems with sleep, agitation, aggression, and feeding [52].

Improvements in activities of daily living were also reported for patients with stroke, COPD, or heart failure at 6-month follow-up [25]. Nutritional status improved for adult patients with acute decompensation of chronic heart failure [49], and fewer patients with dementia assigned to HBH had problems with feeding [52].

The assessment of physical functional capacity for depression and anxiety did not differ between HBH and usual care due to insufficient evidence between groups for most measures [25, 51, 52]. The lack of indicator on functional capacity was also reported in two other reviews [22, 24]. The most recent review emphasized that HBH for end-of-life care may make little difference in functional status, psychological well-being, or cognitive status [24].

A negative indicator on physical functional capacity was reported in patients with stroke mentioning that these patients worsened with HBH intervention compared with treatment in a stroke unit [55].

#### Patient and caregiver satisfaction

In 11 reviews, patient satisfaction was higher with HBH [20, 22, 24, 25, 44, 49–53, 55], two reviews found neutral indicator, citing that patient satisfaction appears to be similar although further robust trials are required [12, 23], and one review found a negative indicator [51].

A considerable proportion of cancer patients, including children and their parents, preferred HBH [50, 53]. Most-valued aspects of HBH are the quality of communication, personal care received [25], frequent and timely visits, and close attention to details [51].

The negative indicator reported concerned women recovering from a hysterectomy and allocated to HBH who had to resume parental responsibilities before being well enough [51].

The satisfaction of informal caregivers was a new indicator documented in the dimension patient and caregiver satisfaction of the NCPF. Four reviews demonstrated positive indicators [20, 25, 50, 52], three showed neutral indicators [22, 25, 51], and two showed negative indicators [25, 44].

HBH increased caregiver satisfaction compared to conventional hospital care [20, 25, 44, 50, 52], notably by lowering relatives’ stress [25], but did not affect carer burden [20] and anxiety of parents of children in HBH [50]. The caregivers reported that although hospital would potentially relieve them from caring, the upheaval of visiting hospital and the accompanying anxiety was a less satisfactory option [25].

However, the results on satisfaction are still uncertain, according to the weak evidence found in three reviews [22, 25, 51], especially for caregivers of patients in end-of-life care. Caregivers expressed lower levels of satisfaction with HBH, compared with hospital care, and experienced lower morale if the participant survived more than 30 days. There was also little or no difference for caregiver bereavement response 6 months following patient’s death [25, 44].

#### Joint contribution of nursing with other care

Seven reviews reported neutral indicators of HBH on mortality at 3- to 6-month follow-up [22, 24–26, 49, 51, 55]. However, positive indicators were described in five reviews that found a tendency to decrease mortality within 2 to 6 months favoring HBH in the middle age group [12, 20, 23, 52], and a reduction in mortality for patients with heart failure compared to conventional hospitalization [54].

HBH indicators on hospital readmission were neutral in seven reviews, as no strong evidence was found on the rate of readmission [22, 25, 26, 49, 51, 52, 55]. However, four reviews mentioned positive indicators with evidence of moderate quality related to the reduction of readmission for HBH patients compared to conventional hospitalization [12, 20, 23, 54], notably in patients with heart failure and COPD [54].

The difference in length of hospital stay varied among reviews, showing a reduction between 4 to 14 days [20, 24, 55]. The positive indicators of HBH on length of stay were documented in six reviews [22–24, 51, 52, 54], two reviews found neutral indicators due to the heterogeneity of the data [25, 55], and a negative indicator was reported in one review [49].

HBH reduced the length of stay for patients with a mix of conditions [22–24, 51, 52, 54]: COPD [54], stroke [22–24, 52], early discharge of patients following elective surgery [51]. However, the total period of care tends to be longer according to one review [23]. Another review showed a significantly longer length of stay in the HBH intervention, but this indicator might be due to the heterogeneity of the data [49].

With respect to survival, a new indicator identified in the dimension joint contribution of nursing with other care, one review found neutral indicator on survival time for HBH end-of-life care [25].

### Overview of the facilitators and barriers to the implementation of HBH model

In total, 41 distinct facilitators and barriers to implementing HBH model were identified and classified in the different categories of factors from the extraction grid. Among these elements, 35 (85%) were classified as facilitators for implementation of HBH and six (15%) as barriers. The complete list of factors can be found in Table 4.

**Table 4:** Frequency of factors identified as facilitators or barriers to the implementation of the HBH model

Factor	No. of systematic reviews and meta-analyses		Example of quotes
	No. of barriers	No. of facilitators	
**1. Factors related to hospital at home (HBH) characteristics**			
1.1 Characteristics of innovation	2	3	Individual’s home situation, social support networks [55]. Nursing care which is only available for the last 2 weeks of life [24].
1.2 Patient empowerment	-	3	Patient and carer education for the recognition and management of acute exacerbation of chronic obstructive pulmonary disease [23]. Self-management education provided at home [49].
**2. Individual factors: knowledge, attitude, and socio-demographic characteristics**			
2.1 Confidence in HBH developer or vendor	1	-	Patients refused HBH due to lack of confidence and were admitted to hospital [52].
2.2 Autonomy	-	1	Differences were reported for patients’ preferred place of care, with each group of patients preferring care at home [51].
2.3 Sociodemographic characteristics	-	1	Strong evidence that patients aged 75 and over may be safely included in early supported discharge (ESD) and hospital at home (HAH) schemes. Most patients hospitalized with acute exacerbation of chronic obstructive pulmonary disease are elderly [23].
**3. External factors: human environment**			
3.1 Patient and health professional interaction	1	-	Miscommunication in teaching the parents [50].
**4. External factors: organizational environment**			
**4.1 Internal environment**			
**4.1.1 Characteristics of the structure of work**			
4.1.1.1 Practice size	-	1	Nursing care available for 24 h if required [25].
4.1.1.2 Workforce issues (shortage, retention)	1	-	Lack of access to 24-h care [25].
**4.1.2 Nature of work**			
4.1.2.1 Work flexibility	-	1	Evening and night cover was provided by a direct line to medical chest unit or provided by district nurses [26].
**4.1.3 Skills (staff)**			
4.1.3.1 Skill mix	-	11	The service was co-ordinated by a nurse [12, 20, 22, 24–26, 52]; rehabilitation services were coordinated with social care [51]. Nurses with respiratory experience [12, 23] or experience in delivering HAH treatment [23].
4.1.3.2 Multidisciplinary collaboration	-	10	Nurse and medical team (including a physician) [53. Specialist and dedicated nurses, specialist physicians, social worker, dietitian, physiotherapist, occupational therapist (OT), speech therapist, and volunteers [22, 51]. Hospital outreach team, a mix of outreach and community staff, general practitioner, community nursing staff, physiotherapist, OT, social worker, counselor, speech therapist, cultural link worker [25, 52].
**4.1.4 Resources**			
4.1.4.1 Material resources (access to information and communication technology)	-	3	Telephone support [23, 49], oxygen therapy, nebulised bronchodilators, intravenous antibiotics, and steroids [23]. Lab values and ECGs done at home, radiographs and echocardiograms at hospital [49].
4.1.4.2 Human resources (information technology (IT) support, other)	1	1	Staff reported that the service was better staffed than usual after care services [51]. Nurses reported that additional help should have been provided for caregivers looking after the participants and for night nursing [24].

### Factors related to HBH characteristics

The included reviews were conducted in different countries with different healthcare systems. Nonetheless, there were some important common features about definition of HBH, which included replacement of both acute and subacute hospitalization [20, 22] in complex patients with a high degree of comorbidity [53, 54] and different intensities of home-based care [20, 49]; care being coordinated in each of the schemes by a multidisciplinary team, home visits, provision of 24-h cover if required, with access to a doctor [12, 20, 23–25, 49, 52, 53] and monitoring, diagnostic testing, home nursing care for the administration of IV medications [49, 53], and a safe home environment [12, 20, 23, 25, 49, 52].

Regarding the factors related to the characteristics of HBH, a total of eight elements pertain to this category, with two of them identified as barriers and six as facilitators. The most recurrent adoption factor was HBH characteristics, with five extracted elements. It was seen as a facilitator in three reviews [20, 23, 55] and as a barrier in two reviews [23, 25]. The configuration of this innovation was characterized mainly by the condition of the individual’s home and the social support networks existing in the HBH model [55] during the day and night [23]. Length of HBH stay was also considered a barrier in HBH model with limited duration [20]. In this context, nursing care available only in the last 2 weeks of life [25] and the heterogeneity of the level of clinical and social support provided in HBH [23] were two shortcomings in the HBH models.

Patient empowerment was mentioned as a facilitator in three reviews [23, 25, 49]. The educational component on self-management provided at home instrumented participants and their families to identify care goals and expected course of disease and outcomes, as well as the probability of success of various treatments [23, 25, 49].

#### Individual factors: knowledge, attitude, and socio-demographic characteristics

Individual factors represented three of the elements identified in the review, two as facilitators and one as a barrier. Only factors related to patients were underlined: confidence in HBH developer or provider, autonomy (health empowerment), and socio-demographical characteristics. The patient’s preferred place of care was the home, which facilitated the implementation of the HBH model [51], with strong evidence on the applicability and safety of the HBH model to the predominant characteristics of patients, aged 75 years or older [23]. Only one individual factor was identified as a barrier in HBH, namely lack of confidence in HBH developer, because a small proportion of patients refused HBH and were admitted to conventional hospital [52].

#### External factors: human environment

External factors related to the human environment refer to the clinical team and their interactions with patients. The only factor identified in this category as a barrier for HBH in children was the miscommunication in teaching parents that affected the patient and health professional interaction [50].

#### External factors: internal organizational environment

Most of the elements reported in the reviews belong to this category, with 27 considered as facilitators and 2 as barriers.

The two most common facilitating factors identified were skill mix [12, 20, 22–26, 49, 51–53] and multidisciplinary collaboration [20, 22–26, 49, 51–53]. About skill mix, the HBH model coordinated by nurses was seen as a facilitator [12, 20, 22, 24–26, 52] as well as rehabilitation services that were coordinated with social care [51]. In relation to nursing skills, HBH was facilitated when nurses were specialists [49, 51] or had experience with respiratory care [12, 23, 25], administration of antineoplastic drugs [53], palliative care [24], and HBH [23].

Multidisciplinary teams are a key facilitator in HBH models. Such teams could include nurses and medical teams (including specialist physicians and family physicians) [20, 22–25, 49, 51–53], social care workers, dietitians, physiotherapists, occupational therapists, speech therapists, pharmacists, psychologists [22–26, 49, 51, 52], volunteers [22, 25, 51], palliative care consultants, nutritionists [24], hospital outreach team, community staff, counselor, and cultural link worker [25, 52].

Characteristics of the structure of work related to practice size [25] and nature of work, specifically the work flexibility for evening and night cover of nursing team [26], were identified as facilitators for HBH. Likewise, provision of material resources (for example, telephone support [23, 25, 49], oxygen therapy, nebulised bronchodilators, intravenous antibiotics and steroids [23], laboratory tests, and electrocardiogram done at home [49]) was also seen as potential facilitators for HBH model implementation.

Two perceived barriers were workforce issues related to reduce nursing staff, especially the night staff, and lack of human resources for 24-h care. The heterogeneous profile of the included patients also required training for nurses, especially for daily home visits. Team composition of the HBH models was heterogeneous and there was a lack of data on who was responsible for each care delivery [24].

## Discussion

### Summary of main results

This systematic review of reviews provides a mapping of the indicators (positive, negative, or neutral indicators) of the HBH model, categorized by the dimensions of nursing care, as well as the factors identified as barriers or facilitators to its implementation.

With respect to the indicators of HBH model, our review corroborates the positive clinical and economic indicators previously documented since 1998. The main contribution of this review is to map these indicators according to the NCPF, which provides a structured approach to analyze how the HBH model works to produce the identified outcomes.

Moreover, our work provides a first review of the factors that could facilitate or hinder the implementation of the HBH model. Our results show that 13 implementation factors influenced the implementation of the HBH model. Among them, the multidisciplinary collaboration and skill mix of professionals inherent to the internal organizational factors were identified as the main facilitators. Conversely, some characteristics of the HBH model, specifically related to the clinical criteria of eligibility and social situation of patients were identified as barriers to implementation.

Based on the AMSTAR2, the overall quality of the included reviews was good, with a number of high and moderate quality reviews, and only two reviews with important methodological limitations. The lack of systematization of data in these reviews led to a lower quality score on the AMSTAR2 scale because we lacked information for assessing the risk of bias. This result may be related to clinical trials that still lack detailed information on the methods used and present methodological flaws that compromise their internal validity [56, 57].

### Discussion of results with respect to the NCPF

In relation to the first function of the NCPF (acquiring, deploying, and maintaining nursing resources), almost all reviews outlined outcomes linked to a dimension of the NCPF called economic sustainability [36]. The positive indicator related to the potential of HBH model to reduce healthcare spending does not directly reflect the total cost of the resources used, including the direct costs for the health sector and the indirect costs related to the impact on families and society, which makes it difficult to assess the cost-effectiveness [12, 20, 22, 23, 26, 44, 49–52, 55]. Two reviews mentioned the importance of service continuity offered in the HBH model since the variations in the way the service is delivered may also account for differences in cost, specifically in HBH schemes that did not provide 24-h care [22, 51]. Regarding the cost of readmission, Echevarria et al. [23] identified conceptual confusion highlighting the need for further detail on this event for patients returning to the hospital during HBH and whether those patients are readmitted at home during the follow-up period.

With respect to the second NCPF function, nursing processes and professional satisfaction were highlighted in the transformation of nursing resources into nursing services. This function captures the benefits of nurse coordinating care in the HBH model for better patient management, particularly with respect to education including self-management [22, 49, 55]. Another review confirmed the importance of patient/family involvement in self-care [23].

Although the HBH model is considered satisfactory for patients in the view of the professionals involved in hospital care, the perceptions of health care providers need to be explored, especially the professional satisfaction related to the work environment characteristics (perceived autonomy, role tension, collaboration). One review highlights that professional satisfaction may determine the potential for adoption of the HBH model and its effective implementation as an alternative hospital model inserted in the existing primary care services [25].

In the third function of the NCPF, the indicators concern the joint contribution of nursing and other systems aimed at the production of changes in patients’ conditions. The environment was associated with positive indicators on patient quality of life [49, 50] and satisfaction of patients and their families [25, 50–53]. In these studies, the HBH environment was qualified as a family environment adapted to the individual that improved the trust relationship with health professionals, increased autonomy, and improved access to the service [49, 50]. For patients and caregivers, home was considered as the preferred place for treatment and hospitalization. Although patients and family members are satisfied with the HBH model, it is still necessary to investigate in detail the participation and perception of family members associated with home hospitalization, particularly regarding the responsibility and the social aspects involved.

### Discussion of results with respect to the factors associated to implementation of the HBH model

Given the considerable attention that the HBH model receives globally, it seemed important to identify the factors identified as facilitators or barriers to its implementation by healthcare organizations. The main findings of this review point out that several internal factors of the organizational environment and factors related to the characteristics of the HBH model influence the implementation of HBH.

The combination of competency of health professionals [12, 20, 22–26, 49, 51–53] and multidisciplinary collaboration [20, 22–26, 49, 51–53] were seen as two important facilitators to the implementation of the HBH model in the included reviews.

Factors related to the characteristics of the HBH model that influence its implementation include innovation characteristics [20, 23, 24, 55] and patient empowerment [23, 25, 49]. Some important features of the HBH model are coordinated care in each multidisciplinary team, 24-h provision with access to a physician, and a safe home environment.

In fact, the diversity of HBH schemes organized according to the national legislation and health systems of the countries was a barrier considered in two reviews [23, 25] because of the different structures of home hospitalization, including the variation of the size of healthcare teams, follow-up visits, and the provision of social and technological support.

The role of HBH model to support patient empowerment has been mentioned in three reviews [23, 25, 49]. In HBH model, healthcare professionals invested in managing patients and education of patients and families for self-care, supporting the idea of a user-centered approach promoted by the level of care provided and intensity of contact with healthcare professionals.

In addition, the support of technology for management and communication among the professionals of the multidisciplinary team [23, 25, 49] and clinical support of HBH patients [23, 49] were also identified in some reviews, albeit less frequently. Although technological resources were a facilitator in the implementation of the HBH model, only a few studies highlighted the need for accessibility to mobile technologies such as telephones and diagnostic equipment.

## Agreements and disagreements with other systematic reviews

Our systematic review of reviews focused on bringing together the scientific evidence on the HBH model published over the last two decades.

We have identified a systematic review of systematic reviews by Conley et al. [58] through our research strategy. Unlike our systematic review, Conley et al. [58] did not focus solely on HBH but examined systematic reviews of alternative management strategies to hospital inpatient unit, including outpatient management, rapid diagnosis units, observation unit, and HBH. Of the 25 systematic reviews selected by Conley et al. [58], only six reviews were related to HBH and of these, four systematic reviews [12, 20, 49, 51] were included in our review. The two others were excluded because they were not systematic reviews.

In relation to clinical outcomes, Conley et al.’s review [58] found positive indicators on cost of resources and patient and caregiver satisfaction for multiple conditions, and neutral indicators on hospital readmission and mortality in HBH management compared with conventional inpatient admission across many acute medical conditions (including heart failure and COPD exacerbations, cellulitis, community-acquired pneumonia, pulmonary embolism, and stroke). They also showed a critical need to determine optimal patient eligibility, and date risk-stratifying algorithms require further evaluation and validation. Diverging from our results, Conley et al. [58] did not find any positive indicators related to additional patient outcomes (functional ability, quality of life, or disease-specific outcomes) in HBH.

## Study strengths and limitations

This systematic review of reviews has many strengths. Firstly, a comprehensive search strategy was developed and implemented in partnership with a health information specialist. Secondly, findings that emerge from the analysis of reviews focus on the evaluation of the indicators of HBH models and the factors influencing their implementation in the form of a narrative synthesis, rather than from the analysis of individual studies. Third, the data extraction process was done with the use of the NCPF and a comprehensive grid of barriers and facilitators to implementation, which supported the organization and the analysis of findings. Moreover, most of the reviews included in this review had a good quality according to the AMSTAR2. However, AMSTAR2 is not intended for the assessment of mixed methods systematic reviews that include qualitative studies, this being one of the limitations of our study.

Some limitations have also been identified. We were limited by the information provided by the reviews authors, which also highlighted a great heterogeneity of HBH schemes in terms of definition, population, interventions, and variety of outcomes. It must be emphasized that some indicators may be overestimated in this review because they come from the same primary studies that have been compiled in different systematic reviews. Despite these limitations, our synthesis contributes to the knowledge base on the HBH model, the facilitating and limiting factors of its implementation, together with the indicators of this model of care in the organization of services, as well as at the clinical, economic, political, and social levels.

## Conclusions

Many systematic reviews have been published on home-based hospitalization, indicating the growing interest in evaluating this intermediary resource on the health services network, specifically the impact on patients, their families, and society.

Our findings provide a mapping of the indicators of the HBH model, categorized by the dimensions of nursing care, as well as the factors identified as barriers or facilitators to its implementation.

The indicators according to the NCPF identified in studies on the implementation of this care model totaled 26 indicators related to nursing care in relation to the cost of resources, management of problems and symptoms, comfort and quality of life, cognitive and psychosocial functional capacity, patient and caregiver satisfaction, hospital mortality, readmissions, and length of stay. Among these, six new indicators were discovered in the analysis of the indicators of the HBH model, namely use of hospital beds, new emergency consultations, and use of healthcare services as indicators of resources of cost, and bowel complications, caregiver satisfaction, and survival time as indicators of change in the patient’s condition. These new indicators provide additional information in the evaluation of the total cost of resources used in the HBH model, including direct costs and indirect costs, and in the evaluation of the production of changes in patients’ conditions based on the joint contribution of nursing and other linked systems in this care model. However, it is still necessary to investigate in detail the cost-effectiveness of the HBH model and the participation and perception of patients and family members associated with home hospitalization.

Regarding the factors that influenced the implementation of the HBH model by health organizations, our review identified 13 factors related mainly to internal organizational factors (multidisciplinary collaboration and skill mix of professionals). Two of these facilitators were seen as important for the implementation of the HBH model in the included reviews, specifically the combination of competence of health professionals and the coordinated care in each multidisciplinary team in home hospitalization. The main barriers were linked to characteristics of the HBH, specifically eligibility criteria (complexity and social situation of the patient) because of the different structures of home hospitalization organized according to the national legislation and health systems of the countries. Since the multidisciplinary collaboration and the skill mix of professionals were found as internal organizational facilitating factors, the limited and transitory approach to the use of technologies in the implementation of the HBH model presented in the systematic reviews analyzed call for further investigation.

We suggest that the use of technologies in the implementation of the HBH model must be addressed in detail with respect to continuity of care and interprofessional collaborations centered on patients and their families, according to the nature of the experience, interests and needs to define the information, and communication technologies interventions necessary for the HBH model.

Although only three reviews included qualitative studies [53–55], we have identified evidence that valued the description of the process of organizing this alternative delivery model to hospital care, highlighting the facilitators and barriers associated with the successful implementation and use of this model of care. Our review shows that the documented indicators are directly related to changes in patient condition and were identified in intermediary resources such as HBH inserted in the care and service trajectory with an impact on structure, process, and results, taking into account the influence of external and internal factors. However, evidence is lacking regarding many outcomes, notably safety and burden on family/informal caregivers.

## Figures and Tables

**Fig. 3:**
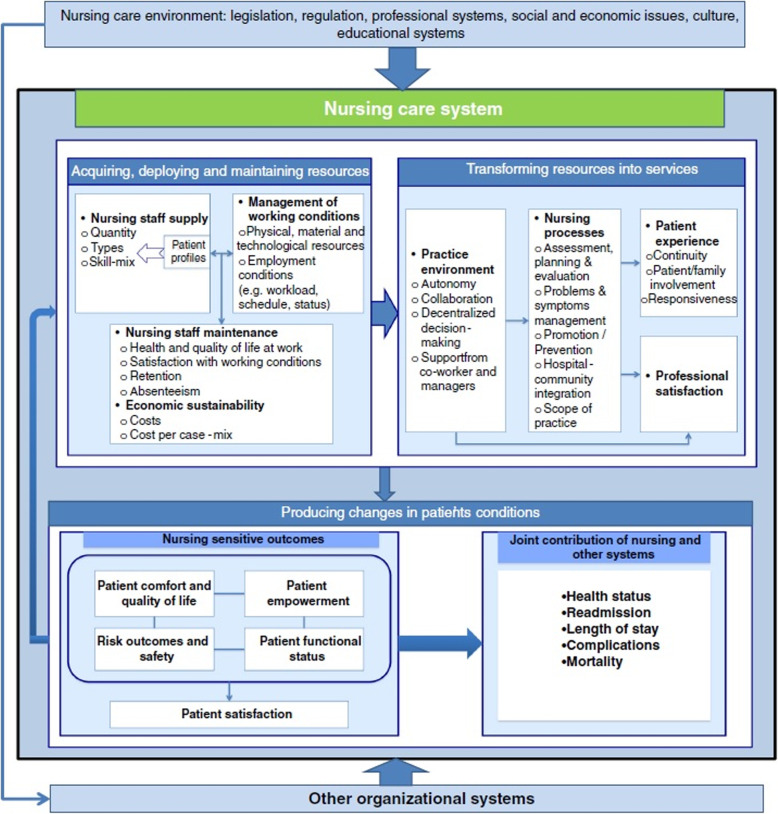
The nursing care performance framework by Dubois* [36]. Legend: asterisk indicates that use and reproduction of this figure was authorized by the author

## Data Availability

All data generated or analyzed during this study are included in this published article and its supplementary information files.

## Appendix 2. Search strategies

**Table 5:** Medline (OVID)

Search	Query	Results
**#**1	(hospital adj2 home).tw.	3707
**#**2	home based versus hospital based.tw.	14
**#**3	home hospitalization.tw.	133
**#**4	exp Home Care Services/	44,262
**#**5	exp Hospitalization/	205,749
**#**6	4 and 5	4826
**#**7	1 or 2 or 3 or 6	8068
**#**8	Meta-Analysis as Topic/	16,136
**#**9	meta analy$.tw.	125,472
**#**10	metaanaly$.tw.	1822
**#**11	Meta-Analysis/	87,336
**#**12	(systematic adj (review$1 or overview$1)).tw.	119,387
**#**13	exp Review Literature as Topic/	9820
**#**14	or/8-13	227,170
**#**15	cochrane.ab.	60,038
**#**16	embase.ab.	63,584
**#**17	(psychlit or psyclit).ab.	909
**#**18	(psychinfo or psycinfo).ab.	22,332
**#**19	(cinahl or cinhal).ab.	20,527
**#**20	science citation index.ab.	2751
**#**21	bids.ab.	451
**#**22	cancerlit.ab.	623
**#**23	or/15-22	104,043
**#**24	reference list$.ab.	15,147
**#**25	bibliograph$.ab.	15,647
**#**26	hand-search$.ab.	5844
**#**27	relevant journals.ab.	1042
**#**28	manual search$.ab.	3685
**#**29	or/24-28	37,062
**#**30	selection criteria.ab.	26,761
**#**31	data extraction.ab.	16,097
**#**32	30 or 31	40,796
**#**33	Review/	2,369,814
**#**34	32 and 33	27,208
**#**35	Comment/	713,540
**#**36	Letter/	983,939
**#**37	Editorial/	455,744
**#**38	animal/	6,190,908
**#**39	human/	17,017,485
**#**40	38 not (38 and 39)	4,414,780
**#**41	or/35-37,40	5,976,817
**#**42	14 or 23 or 29 or 34	273,439
**#**43	42 not 41	259,332
**#**44	7 and 43	**239**

**Table 6:** CINAHL Plus with Full Text

Search	Query	Results
S11	S5 AND S10	**306**
S10	S6 OR S7 OR S8 OR S9	126,202
S9	systematic N2 (review or overview)	92,36
S8	(MH “Literature Review+”)	62,327
S7	Meta analys* OR Metaanaly*	56,065
S6	(MH “Meta Analysis”)	31,89
S5	S1 OR S2 OR S3 OR S4	7688
S4	(MH “Home Health Care”) AND (MH “Hospitalization”)	590
S3	TI Home hospitalization OR AB Home hospitalization	3477
S2	TI Home-based versus hospital-based OR AB Home-based versus hospital-based	24
S1	TI hospital N2 home OR AB hospital N2 home	4222

**Table 7:** Embase

Search	Query	Results
**#**40	# 32 OR #39	**361**
**#**39	#33 OR #34 OR #35 OR #38	9305
**#**38	#36 AND #37	4243
**#**37	‘hospitalization’/de	302,681
**#**36	‘home care’/exp	66,443
**#**35	‘home-based versus hospital based’:ab,ti	18
**#**34	‘home hospitalization’:ab,ti	199
**#**33	(‘hospital’ NEAR/2 ‘home’):ab,ti	5258
**#**32	#31 NOT #30	372,637
**#**31	#4 OR #13 OR #19 OR #24	387,258
**#**30	#25 OR #26 OR #29	2,985,597
**#**29	#27 NOT (59 AND #28)	1,394,722
**#**28	‘human’/de	19,205,255
**#**27	‘animal’/de	1,829,542
**#**26	‘letter’:it OR ‘letter’/de	1,013,470
**#**25	‘editorial’:it OR ‘editorial’/de	602,895
**#**24	#22 AND #23	24,469
**#**23	‘review’/de OR review:it	2,445,565
**#**22	#20 OR #21	48,611
**#**21	‘selection criteria’:ab	31,177
**#**20	‘data extraction’:ab	19,337
**#**19	#14 Or #15 OR #16 OR #17 OR #18	42,960
**#**18	‘relevant journals’:ab	1210
**#**17	‘manual search*’:ab	4282
**#**16	‘hand-search*’:ab	6862
**#**15	‘bibliograph*’:ab	19,517
**#**14	‘reference lists’:ab	15,852
**#**13	#5 OR #6 OR #7 OR #8 OR #9 Or #10 OR #11 OR #12	123,233
**#**12	bids:ab	564
**#**11	‘science citation index’:ab	3113
**#**10	cinahl:ab OR cinalh:ab	22,329
**#**9	psychinfo:ab OR psycinfo:ab	19,854
**#**8	psychlit:ab OR psyclit:ab	977
**#**7	embase:ab	78,585
**#**6	cochrane:ab	76,068
**#**5	cancerlit:ab	708
**#**4	#1 OR #2 OR #3	340,912
**#**3	(systematic NEAR/1 (review* OR overview*)):ab,ti	143,580
**#**2	((meta NEAR/1 analy*):ti,ab) OR metaanalys*:ti,ab	162,940
**#**1	‘systematic review’/de OR ‘meta analysis’/exp OR ‘meta analysis (topic)’/de	272,499

**Table 8:** **Cochrane**

Search	Query	Results
**#**1	hospital near/2 home:ti,ab,kw	1004
**#**2	home hospitalization:ti,ab,kw	1880
**#**3	Home-based versus hospital-based :ti,ab,kw	60
**#**4	#1 or #2 or #3	**2650**
	Cochrane Database of Systematic Reviews (CDSR)	**117**
	Database of Abstracts of Reviews of Effects (DARE)	**46**

## Appendix 3

**Table 9:** Preferred Reporting Items for Systematic review and Meta-Analysis extension for Network Meta-Analyses (PRISMA-NMA) checklist [31]

Section/topic	#	Checklist item	Reported on page #
**Title**			
Title	1	Identify the report as a systematic review incorporating a network meta-analysis (or related form of meta-analysis).	1
**Abstract**			
Structured summary	2	Provide a structured summary including, as applicable: Background: main objectives. Methods: data sources; study eligibility criteria, participants, and interventions; study appraisal; and synthesis methods, such as network meta-analysis. Results: number of studies and participants identified; summary estimates with corresponding confidence/credible intervals; treatment rankings may also be discussed. Authors may choose to summarize pairwise comparisons against a chosen treatment included in their analyses for brevity. Discussion/conclusions: limitations; conclusions and implications of findings. Other: primary source of funding; systematic review registration number with registry name.	2,3
**Introduction**			
Rationale	3	Describe the rationale for the review in the context of what is already known, including mention of why a network meta-analysis has been conducted.	3, 4
Objectives	4	Provide an explicit statement of questions being addressed, with reference to participants, interventions, comparisons, outcomes, and study design (PICOS).	5
**Methods**			
Protocol and registration	5	Indicate whether a review protocol exists and if and where it can be accessed (e.g., Web address); and, if available, provide registration information, including registration number.	6
Eligibility criteria	6	Specify study characteristics (e.g., PICOS, length of follow-up) and report characteristics (e.g., years considered, language, publication status) used as criteria for eligibility, giving rationale. Clearly describe eligible treatments included in the treatment network and note whether any have been clustered or merged into the same node (with justification).	6, 7
Information sources	7	Describe all information sources (e.g., databases with dates of coverage, contact with study authors to identify additional studies) in the search and date last searched.	7
Search	8	Present full electronic search strategy for at least one database, including any limits used, such that it could be repeated.	54
Study selection	9	State the process for selecting studies (i.e., screening, eligibility, included in systematic review, and, if applicable, included in the meta-analysis).	7
Data collection process	10	Describe method of data extraction from reports (e.g., piloted forms, independently, in duplicate) and any processes for obtaining and confirming data from investigators.	8
Data items	11	List and define all variables for which data were sought (e.g., PICOS, funding sources) and any assumptions and simplifications made.	6
Geometry of the network	S1	Describe methods used to explore the geometry of the treatment network under study and potential biases related to it. This should include how the evidence base has been graphically summarized for presentation, and what characteristics were compiled and used to describe the evidence base to readers.	8
Risk of bias in individual studies	12	Describe methods used for assessing risk of bias of individual studies (including specification of whether this was done at the study or outcome level), and how this information is to be used in any data synthesis.	This is not applicable
Summary measures	13	State the principal summary measures (e.g., risk ratio, difference in means). Also describe the use of additional summary measures assessed, such as treatment rankings and surface under the cumulative ranking curve (SUCRA) values, as well as modified approaches used to present summary findings from meta-analyses.	This is not applicable
Planned methods of analysis	14	Describe the methods of handling data and combining results of studies for each network meta-analysis. This should include, but not be limited to: handling of multigroup trial, selection of variance structure, selection of prior distributions in Bayesian analyses, and assessment of model fit.	This is not applicable
Assessment of inconsistency	S2	Describe the statistical methods used to evaluate the agreement of direct and indirect evidence in the treatment network(s) studied. Describe efforts taken to address its presence when found.	This is not applicable
Risk of bias across studies	15	Specify any assessment of risk of bias that may affect the cumulative evidence (e.g., publication bias, selective reporting within studies).	This is not applicable
Additional analyses	16	Describe methods of additional analyses if done, indicating which were prespecified. This may include, but not be limited to, the following: sensitivity or subgroup analyses; meta-regression analyses; alternative formulations of the treatment network, and use of alternative prior distributions for Bayesian analyses (if applicable).	This is not applicable
**Results**			
Study selection	17	Give numbers of studies screened, assessed for eligibility, and included in the review, with reasons for exclusions at each stage, ideally with a flow diagram.	9
		Provide a network graph of the included studies to enable visualization of the geometry of the treatment network.	
Presentation of network structure	S3	Provide a network graph of the included studies to enable visualization of the geometry of the treatment network.	67
Summary of network geometry	S4	Provide a brief overview of characteristics of the treatment network. This may include commentary on the abundance of trials and randomized patients for the different interventions and pairwise comparisons in the network, gaps of evidence in the treatment network, and potential biases reflected by the network structure.	11, 76
Study characteristics	18	For each study, present characteristics for which data were extracted (e.g., study size, PICOS, follow-up period) and provide the citations.	11, 76
Risk of bias within studies	19	Present data on risk of bias of each study and, if available, any outcome level assessment.	This is not applicable
Results of individual studies	20	For all outcomes considered (benefits or harms), present, for each study: (1) simple summary data for each intervention group, and (2) effect estimates and confidence intervals. Modified approaches may be needed to deal with information from larger networks.	9–11, 76
Synthesis of results	21	Present results of each meta-analysis done, including confidence/credible intervals. In larger networks, authors may focus on comparisons versus a particular comparator (e.g., placebo or standard care), with full findings presented in an appendix. League tables and forest plots may be considered to summarize pairwise comparisons. If additional summary measures were explored (such as treatment rankings), these should also be presented.	15-35
Exploration for inconsistency	S5	Describe results from investigations of inconsistency. This may include such information as measures of model fit to compare consistency and inconsistency models, *P* values from statistical tests, or summary of inconsistency estimates from different parts of the treatment network.	39–41
Risk of bias across studies	22	Present results of any assessment of risk of bias across studies for the evidence base being studied.	This is not applicable
Results of additional analyses	23	Give results of additional analyses, if done (e.g., sensitivity or subgroup analyses, meta-regression analyses, alternative network geometries studied, and alternative choice of prior distributions for Bayesian analyses).	This is not applicable
**Discussion**			
Summary of evidence	24	Summarize the main findings, including the strength of evidence for each main outcome; consider their relevance to key groups (e.g., health care providers, researchers, and policymakers).	36-39
Limitations	25	Discuss limitations at study and outcome level (e.g., risk of bias), and at review level (e.g., incomplete retrieval of identified research, reporting bias). Comment on the validity of the assumptions, such as transitivity and consistency. Comment on any concerns regarding network geometry (e.g., avoidance of certain comparisons).	40–41
Conclusions	26	Provide a general interpretation of the results in the context of other evidence and implications for future research.	41–42
**Funding**			
Funding	27	Describe sources of funding for the systematic review and other support (e.g., supply of data); role of funders for the systematic review. This should also include information regarding whether funding has been received from manufacturers of treatments in the network and/or whether some of the authors are content experts with professional conflicts of interest that could affect use of treatments in the network.	43

## Appendix 4

**Table 10:** Primary studies of HBH included in systematic reviews and meta-analyses

Primary studies	Systematic reviews and meta-analyses															Total
	[53]	[54]	[22]	[55]	[24]	[25]	[23]	[49]	[20]	[12]	[50]	[51]	[52]	[26]	[44]	
**Cocquyt, 2016**	x															1
**Dey, 2016**			x									x				2
**Karlsson, 2016**			x													1
**Touati, 2016**	x															1
**Lal, 2015**	x															1
**Crisp, 2014**	x															1
**Ince, 2014**			x													1
**García-Soleto, 2013**				x												1
**Lasalle, 2016**	x															1
**Lau, 2013**				x												1
**Tibaldi, 2013**			x													1
**Vianello, 2013**						x										1
**Lüthi, 2012**	x															1
**Utens, 2012**			x				x									2
**Andrei, 2011**						x										1
**Aujesky 2011**									x							1
**Crilly, 2011**				x												1
**Talcott 2011**						x			x							2
**Meenaghan, 2010**	x															1
**Otero, 2010**				x					x							2
**Frick, 2009**				x												1
**Leff, 2009**				x												1
**Mendoza, 2009**		x		x		x		x	x							5
**Rodríguez-Cerrillo, 2009**				x												1
**Tibaldi, 2009**				x		x		x	x							4
**Hall, 2008**	x															1
**Patel, 2008**				x				x	x							3
**Rada, 2008**			x													1
**Ricauda, 2008**									x							1
**Ricauda, 2008**				x		x	x			x						4
**Brumley, 2007**					x											1
**Nissen, 2007**							x			x						2
**Caplan, 2006**			x									x				2
**Stevens, 2006**											x					1
**Vergnenègre, 2006**	x															1
**Carratalà, 2005**				x					x							2
**Corwin, 2005**						x			x				x			3
**Diaz Lobato, 2005**		x	x						x							3
**Harris, 2005**			x			x						x	x			4
**Leff, 2005**				x												1
**Richards, 2005**						x							x			2
**Rodríguez-Cerrillo, 2005**				x												1
**Askim, 2004**			x									x				2
**Booth, 2004**			x									x				2
**Cunliffe, 2004**			x									x				2
**Donnelly, 2004**			x									x				2
**Ricauda, 2004**						x							x			2
**Tibaldi, 2004**						x			x				x			3
**Anderson, 2003**	x															1
**de Zuazu, 2003**								x								1
**Hernandez, 2003**		x							x	x				x		4
**Virally, 2003**	x															1
**Bautz-Holter, 2002**			x									x				2
**Corrie, 2013**	x															1
**Crotty, 2002**			x									x				2
**Miano, 2002**											x					1
**Ojoo, 2002**			x				x		x	x		x		x		6
**Remonnay, 2002**	x															1
**Farrero, 2001**		x														1
**Nicholson, 2001**						x			x	x			x	x		5
**Suwenwela, 2001**			x									x				2
**Anderson, 2000**			x									x				2
**Bechich, 2000**								x								1
**Borras, 2001**	x															1
**Cotton, 2000**			x				x		x	x		x		x		6
**Davies, 2000**						x	x		x	x			x	x		6
**Grande, 2000**					x											1
**Indredavik, 2000**			x									x				2
**Jordhøy, 2000**					x											1
**Kalra, 2000**				x		x							x			3
**King, 2000**	x															1
**Mayo, 2000**			x									x				2
**Palmer Hill, 2000**			x									x				2
**Rischin, 2000**	x															1
**Skwarska, 2000**			x				x		x	x		x		x		6
**Caplan, 1999**						x			x				x			3
**Wilson, 1999**						x			x				x			3
**Richards, 1998**			x						x			x				3
**Shepperd 1998**			x						x			x		x		4
**Widén Holmqvist, 1998**			x									x				2
**Holdsworth, 1997**											x					1
**Rodgers, 1997**			x									x				2
**Rudd, 1997**			x									x				2
**Koopman, 1996**									x							1
**Levine, 1996**									x							1
**Close, 1995**											x					1
**Donald, 1995**			x									x			x	3
**Martin, 1994**			x									x			x	3
**Hughes, 1992**					x										x	2
**Melin, 1992**									x							1
**Payne, 1992**	x															1
**Lange, 1988**											x					1
**Adler, 1978**			x									x			x	3
**Hill, 1978**									x							1
**Ruckley, 1978**			x									x			x	3
**Mather, 1976**									x							1
**Total**	**17**	**4**	**32**	**15**	**4**	**16**	**7**	**5**	**26**	**8**	**5**	**26**	**10**	**7**	**5**	**187**

## Appendix 5

**Table 11:** Excluded articles

Justification	References of excluded articles
**Old version**	Ram FSF, Wedzicha JA, Wright JJ, Greenstone M. Hospital at home for acute exacerbations of chronic obstructive pulmonary disease. Cochrane Database of Systematic Reviews 2003, Issue 4. Art. No.: CD003573. DOI: 10.1002/14651858.CD003573. Update [12]
	Ram FSF, Wedzicha JA, Wright JJ, Greenstone M, Lasserson TJ. Hospital at home for acute exacerbations of chronic obstructive pulmonary disease. Cochrane Database System Rev 2009 (4). 20 OCT 2003. DOI: 10.1002/14651858.CD003573 Update [12]
	Conley J, O’Brien CW, Leff BA, et al. Alternative strategies to inpatient hospitalization for acute medical conditions: a systematic review. JAMA Intern Med 2016;176:1693–702.doi:10.1001/jamainternmed.2016.5974. Update [58]
	Shepperd S, Iliffe S. Hospital at home versus in-patient hospital care. Cochrane database of systematic reviews (online) 2001; null(3): CD000356. Update [22]
	Shepperd S, Iliffe S. Cochrane reviews. Hospital at home versus in-patient hospital care. Nursing Times. 2001;97(38):37. Update [22]
	Parkes J, Shepperd S. Cochrane reviews. Discharge planning from hospital to home. Nursing Times. 2001;97(37):42. Update [22]
	Shepperd S, Iliffe S. Hospital at home versus in-patient hospital care. Cochrane Database of Systematic Reviews 2005, Issue 3. Art. No.: CD000356. DOI: 10.1002/14651858.CD000356.pub2. Update [22]
	Langhorne P, Dennis M, Kalra L, Shepperd S, Wade D, Wolfe CDA. Services for helping acute stroke patients avoid hospital admission. Cochrane Database of Systematic Reviews 1999, Issue 3. Art. No.: CD000444. DOI: 10.1002/14651858.CD000444. Update [52]
**Commentary**	Without author. ‘Hospital at home’ schemes are as safe as inpatient care for people with exacerbated chronic obstructive pulmonary disease (COPD). Evidence-Based Healthcare and Public Health. 2005;9(1):46-7.
	Inpatient and hospital-at-home care: the same outcomes? Nursing Times, 104 (48), 29; 2008.
	Grad R. Review: hospital-at-home care for early discharge or admission avoidance does not improve health outcomes. ACP J Club. 2002 Jul-Aug;137:23.
	Jacobs MB. Review: hospital-at-home care does not increase mortality or readmission rates in acute exacerbations of COPD. ACP Journal Club. 2004;140(3):59.
	Reishtein JL. Review: hospital at home is as effective as inpatient care for mortality and hospital readmissions in patients with acute exacerbations of chronic obstructive pulmonary disease. Evidence Based Nursing. 2005;8(1):23.
	Dickson HG. A meta-analysis of “hospital in the home”. Comment. The Medical journal of Australia. 2013;198(4):195.
**No systematic review**	Mas MT, Santaeugènia S. Hospital-at-home in older patients: a scoping review on opportunities of developing comprehensive geriatric assessment based services. Revista Espanola de Geriatria y Gerontologia. 2015;50(1):26-34.
	Messecar D. Review: admission-avoidance hospital-at-home decreases mortality at 6 months but does not differ from inpatient care for readmission. Evidence Based Nursing. 2009;12(3):82.
	Mitre Cotta RM, Suárez-Varela MM, Llopis González A, Cotta Filho JS, Real ER, Días Ricós JA. Home hospitalization: background, current situation, and future prospects. Revista Panamericana de Salud Publica. 2001;10(1):45-55.
**Abstract**	Goossens LMA, Vemer P, Rutten-Van Mölken MPMH. A systematic review of hospital-at-home care: cost savings are overestimated. Value in Health. 2012;15(7):A301.
	Iliffe S, Shepperd S. What do we know about hospital at home? Lessons from international experience. Applied health economics and health policy. 2002;1(3):141-7.
**Analysis**	McCurdy BR. Hospital-at-home programs for patients with acute exacerbations of chronic obstructive pulmonary disease (COPD): an evidence-based analysis. Ontario Health Technology Assessment Series. 2012;12(10):1-65.
**Editorial**	Shepperd S, Cates C. Hospital at home in chronic obstructive pulmonary disease: is it a viable option? Cochrane Database of Systematic Reviews [Internet]. 2012; (5). Available from: http://cochranelibrary-wiley.com/doi/10.1002/14651858.ED000042/abstract.
**Letter**	Caplan GA. A meta-analysis of “hospital in the home”. The Medical journal of Australia. 2013;198(4):195-6.
**Included results of others reviews**	Shepperd S, Wee B, Straus SE. Hospital at home: home-based end of life care. Cochrane Database of Systematic Reviews. 2011(7):CD009231.
**Published the results in another journal**	Shepperd S, Doll H, Angus RM, Clarke MJ, Iliffe S, Kalra L, et al. Avoiding hospital admission through provision of hospital care at home: a systematic review and meta-analysis of individual patient data. CMAJ: Canadian Medical Association Journal. 2009;180(2):175-82.
**Systematic review of systematic reviews included in our study**	Conley J, O'Brien CW, Leff BA, Bolen S, Zulman D. Alternative strategies to inpatient hospitalization for acute medical conditions: a systematic review. JAMA Internal Medicine. 2016;176(11):1693-702.

## Appendix 6

**Table 12:** Characteristics of studies included in detail

Author, year, country	Type of reviews or designs	No. of studies	Study country	Population	Intervention	Outcomes	AMSTAR
**Cool et al.** [53], **2018**, Belgium	Systematic review Mixed	5 RCT* 2 nRCT* 7 single-arm prospective trials 2 qualitative studies 1 retrospective cohort study	European countries, mostly France and United Kingdom (UK) Other studies from: Belgium, Israel, Germany, Ireland, Spain, Switzerland, Sweden, Australia, Canada, and United States of America (USA)	Adult patients	Parenteral cancer drug administration in hospital at home care	Quality of life, patient’s satisfaction, safety, and costs	Moderate quality review
**Corral Gudino et al.** [54], **2017**, Spain	Systematic review Qualitative	21 RCTs, among which 4 RCTs about HBH	Spain	Not specified	Interventions supporting continuity of care, including HBH	Number of readmissions, mortality, or improvement in functional capacity	Moderate quality review
**Goncalves-Bradley et al.** [22], ** 2017**, UK	Systematic review and meta-analysis Quantitative	32 RCTs	A majority of studies are from the UK, Australia, and Norway. Other studies from: Canada, Chile, Italy, New Zealand, Spain, Sweden, Thailand, The Netherlands, and Turkey	Patients aged 18 years and over, acute episode of care	Early discharge hospital at home	Effectiveness and cost of the intervention	High quality review
**Huntley et al.** [55], **2017**, UK	Systematic review Qualitative	10 RCTs 9 nRCTs Among them 11 studies (6 RCTs and 5 nRCTs) about HBH	European countries, principally UK	Patients aged over 65 years at risk of an unplanned admission	Any community-based intervention offered as an alternative to admission to an acute hospital	Reduction in secondary care use, patient-related outcomes, safety, and costs	Moderate quality review
**Shepperd et al.** [24], **2016a**, UK	Systematic review and meta-analysis Quantitative	3 RCTs 1 nRCT	Norway, UK, and USA	People aged 18 years and older, who would otherwise require hospital or hospice inpatient end-of-life care	Home-based end-of-life care	Place of death, unplanned/precipitous admission to or discharge from hospital, control of symptoms, delay in care from point of referral to intervention, participant health outcomes, family- or caregiver-reported symptoms, family or caregiver unable to continue caring, participant’s preferred place of death, health service use, including system and caregiver costs	High quality review
**Shepperd et al.** [25], **2016b**, UK	Systematic review and meta-analysis Quantitative	16 RCTs	Australia, Italy, New Zealand, Romania, Spain, UK, and USA	Patients aged 18 years and over (older patients = 65 years and older). Patients to be clinically stable and not requiring specialist diagnostic investigation or emergency interventions	Hospital at home	Mortality, transfer (or readmission) to hospital, functional status, quality of life or self-reported health status, cognitive function, depression, clinical outcomes, place of residence at follow-up (living in a residential setting), patient satisfaction, caregiver outcomes, health professionals’ views, length of stay in hospital and hospital at home, cost, use of other health services and informal care	High quality review
**Echevarria et al.** [23], **2016**, UK	Systematic review and meta-analysis Quantitative	7 RCTs	UK, Netherlands, Australia, Italy	Patients with acute exacerbation of chronic obstructive pulmonary disease	Early supported discharge (ESD) and hospital at home (HAH)	Structure of ESD/HAH schemes, number of patients experiencing one or more readmissions, mortality and cost, comparing cost across different countries and healthcare structures	Moderate quality review
**Qaddoura et al.** [49], **2015**, Canada	Systematic review and meta-analysis Quantitative	3 RCTs 3 nRCTs among which 5 studies (3 RCTs and 2 nRCTs) are about HBH	Spain, Italy, Sweden	Patients who required hospitalization for decompensated heart failure	Substitutive care models	Mortality, hospital readmissions, other clinical, patient-centered, and cost outcomes	High quality review
**Caplan et al.** [20], **2012**, Australia	Systematic review and meta-analysis Quantitative	61 RCTs among which 26 RCTs about HBH	Countries are not explicitly mentioned	Patients aged > 16 years	Hospital at home care models regardless of temporal-, team- and disease-specific frameworks	Mortality, readmission rates, patient and carer satisfaction, and costs	Moderate quality review
**Jeppesen et al.** [12], **2012**, Norway	Systematic review and meta-analysis Quantitative	8 RCTs	Australia, Denmark, Italy, Spain, UK	Patients with a diagnosis of COPD with an acute exacerbation	Hospital at home care	Readmission rate, mortality, costs and days of care provision	High quality review
**Hansson et al.** [50], **2011**, Denmark	Systematic review Quantitative	1 RCT 1 control group 3 studies had no true control group	USA, Canada, and Italy	Children and adolescents aged 0–18 years with a cancer diagnosis	Medical treatments relevant for childhood cancer provided by hospital- or community-based healthcare professionals who take an active part in the care in the patient’s own home as an alternative to a hospital admission	Children’s physical health, adverse events, parental and child satisfaction, QOL of children and their parents, and costs of using hospital data, questionnaires, or satisfaction surveys	Low quality review
**Shepperd et al.** [51], **2009**, UK	Systematic review and meta-analysis Quantitative	26 RCTs	Countries are not explicitly mentioned	Patients aged 18 years and over (older patients = 65 years and older). People requiring long-term care needs were not included unless they required admission to hospital for an acute episode of care	Early discharge hospital at home	Mortality, readmissions, general and disease-specific health status, functional status, psychological well-being, clinical complications, patient satisfaction, carer satisfaction, carer burden, staff views, discharge destination from hospital at home, length of stay in hospital and hospital at home, cost	Moderate quality review
**Shepperd et al.** [52], **2008**, UK	Systematic Review and Meta-Analysis Quantitative	10 RTCs	Australia, Italy, New Zealand and the UK	Patients aged 18 years and older entered the program directly from the community or from the emergency department	Hospital care at home	Mortality, readmissions or transfers to hospital, general and disease-specific health status, functional status, psychological well-being, clinical complications, patient and caregiver satisfaction, caregiver burden, staff perspectives, place of residence at followup, length of stay and cost	Moderate quality review
**Felix et al.** [26], **2004**, UK	Systematic review and meta-analysisQuantitative	7 RTCs	Spain, Australia (not explicitly mentioned other countries included)	Adult patients attending an emergency department with an acute exacerbation within 72 h of presenting to the department and after an	Hospital at home schemes	Mortality and readmission	Moderate quality review
**Shepperd et al.** [44], **1998**, UK	Systematic review Quantitative	5 RCTs	UK, USA	Patients aged 18 years and over needing treatment during an acute episode of care	Hospital at home care	Mortality, clinical complications, re-admissions, costs, hospital days saved from the provision of hospital at home, discharge destination from hospital at home, functional status, psychological well-being, patient satisfaction, and carer satisfaction	Low quality review

*Legend: Randomized controlled trial (RTC) and Non-randomized controlled trial (nRTC)

## Appendix 7

**Table 13:** Strength of evidence of reported indicators of HBH model using GRADE [46].

Effects	[24]	[25]	[22]	[12]
**Readmission**	Moderate	-	Low	Moderate
**Mortality**	Moderate	-	Moderate	Moderate
**Living in an institutional setting**	Low	-	Low	-
**Hospital and hospital at home length of stay**	Low	-	Low	-
**Place of death (home)**	-	High	-	-
**Admission to hospital**	-	Moderate	-	-
**Patient satisfaction**	Low	Low	Low	Low
**Caregiver burden**	-	Low	-	-
**Carer satisfaction**	-	-	-	Very low
**Health service cost**	Low	Low	Very low	Very low
**Quality of life**	-	-	-	Very low

Legend: GRADE Working Group grades of evidence: High certainty: further research is very unlikely to change our confidence in the estimate of effect. Moderate certainty: further research is likely to have an important impact on our confidence in the estimate of effect and may change the estimate. Low certainty: further research is very likely to have an important impact on our confidence in the estimate of effect and is likely to change the estimate. Very low certainty: we are very uncertain about the estimate

## Abbreviations

AMSTAR 2: Assessing the Methodological quality of Systematic Reviews–update
CDSR: Cochrane Database of Systematic Reviews
CINAHL: Cumulative Index to Nursing and Allied Health Literature
CIUSSS-CN: Centre intégré universitaire de santé et services sociaux de la Capitale-Nationale
COPD: Chronic obstructive pulmonary disease
DARE: Database of Abstracts of Reviews of Effects
EndNote: Bibliographic reference management software
ESD: Early supported discharge
HAH: Hospital at home
HBH: Home-based hospitalization
HBHC: Hospital-based home care
IT: Information technology
Medline: Medical Literature Analysis and Retrieval System Online
NCPF: Nursing care performance framework
nRTC: Non-randomized controlled trial
PRISMA: Preferred Reporting Items for Systematic Review and Meta-Analysis
PROSPERO: International Prospective Register of Systematic Reviews
QoL: Quality of life
RTC: Randomized controlled trial
